# The lncRNA *PVT1* Contributes to the Cervical Cancer Phenotype and Associates with Poor Patient Prognosis

**DOI:** 10.1371/journal.pone.0156274

**Published:** 2016-05-27

**Authors:** Marissa Iden, Samantha Fye, Keguo Li, Tamjid Chowdhury, Ramani Ramchandran, Janet S. Rader

**Affiliations:** 1 Department of Obstetrics and Gynecology, Medical College of Wisconsin, Milwaukee, WI, United States of America; 2 Department of Pediatrics, Medical College of Wisconsin, Milwaukee, WI, United States of America; National Institute of Technology, Rourkela, INDIA

## Abstract

The plasmacytoma variant translocation 1 gene (*PVT1*) is an lncRNA that has been designated as an oncogene due to its contribution to the phenotype of multiple cancers. Although the mechanism by which *PVT1* influences disease processes has been studied in multiple cancer types, its role in cervical tumorigenesis remains unknown. Thus, the present study was designed to investigate the role of *PVT1* in cervical cancer *in vitro* and *in vivo*. *PVT1* expression was measured by quantitative PCR (qPCR) in 121 invasive cervical carcinoma (ICC) samples, 30 normal cervix samples, and cervical cell lines. Functional assays were carried out using both siRNA and LNA-mediated knockdown to examine *PVT1’*s effects on cervical cancer cell proliferation, migration and invasion, apoptosis, and cisplatin resistance. Our results demonstrate that *PVT1* expression is significantly increased in ICC tissue versus normal cervix and that higher expression of *PVT1* correlates with poorer overall survival. In cervical cancer cell lines, *PVT1* knockdown resulted in significantly decreased cell proliferation, migration and invasion, while apoptosis and cisplatin cytotoxicity were significantly increased in these cells. Finally, we show that *PVT1* expression is augmented in response to hypoxia and immune response stimulation and that this lncRNA associates with the multifunctional and stress-responsive protein, Nucleolin. Collectively, our results provide strong evidence for an oncogenic role of *PVT1* in cervical cancer and lend insight into potential mechanisms by which *PVT1* overexpression helps drive cervical carcinogenesis.

## Introduction

Long noncoding RNAs (lncRNAs) provide important targets for cancer diagnostics and therapeutics due to their critical role in numerous cellular processes such as epigenetic changes, gene enhancer and tumor suppressor activity, and miRNA sequestering. LncRNAs are pervasive in the genome, frequently show cell type- and temporal-specific regulation of gene expression, and can influence many cellular processes via multiple disparate mechanisms [1]. Plasmacytoma variant translocation 1 (*PVT1*) is a highly conserved lncRNA ~50kb downstream of *MYC* that has attracted significant attention from the cancer field due to its frequent co-amplification with *MYC* in several solid tumors [2]. The first studies providing evidence that *PVT1* may contribute to carcinogenesis demonstrated frequent translocations in mouse plasmacytomas [3,4] and human Burkitt’s lymphomas [5–7]. The oncogenic effects of *PVT1* have been further highlighted by more recent studies demonstrating its overexpression and amplification in multiple cancer types [8–17]. More importantly, *PVT1* expression has been significantly correlated with clinical features such as risk, recurrence, and survival in various cancers [8,11–13,18].

Despite the wealth of knowledge regarding the oncogenic properties of *PVT1* in multiple cancers, very little is known about its precise biologic function. In fact, the handful of studies providing *PVT1* mechanistic data exceedingly suggest that it exerts its effects in a cell-type and/or disease-specific manner. For example, *PVT1* function has been attributed to its binding and stabilization of the Myc [19] and Nop2 [17] proteins in breast and hepatocellular carcinoma, respectively. In gastric cancer cells, *PVT1* acts to repress the expression of p15 and p16 via its physical interaction with the polycomb group protein, EZH2 [20]. Also via EZH2 recruitment and regulation of thyroid-stimulating hormone receptor, *PVT1* induces proliferation of thyroid cancer cells [21]. Finally, computational analysis of *PVT1* suggests that it may act via binding and sequestration of mir-200 family members in breast cancer tissue [14]. Due to their complexity and breadth of results, these studies emphasize the importance of disease-specific investigation of *PVT1* mechanisms.

Our interest in *PVT1* originated from our work and others’ demonstrating that it is a frequent site of HPV integration in cervical cancer, further suggesting an important biological function [22–24]. Thus, we sought to better define the role of *PVT1* in cervical carcinogenesis by examining *PVT1* expression patterns in cervical cancer tumors and cell lines and the functional effects of this lncRNA on cancer-related processes such as proliferation, cell motility, apoptosis, and chemoresistance. Finally, because many lncRNAs rely on protein partners to carry out their effects, we employed RNA affinity chromatography and mass spectrometry sequencing to elucidate cervical cancer cell-specific *PVT1* binding partners.

## Methods

The study protocol was approved by the institutional review board of the Medical College of Wisconsin IRB (PRO00011466; Molecular Characterization of Cervical Cancer).

### Cell culture

SiHa (ATCC® HTB35™), HeLa (ATCC® CCL2™), and DoTc2 4510 (ATCC® CRL-7920™) human cervical cancer cell lines and HPV 16 E6/E7-transformed, normal ectocervical cells (Ect1/E6E7; ATCC® CRL-2614™) were obtained from the American Type Culture Collection (ATCC, Manassas, VA). Cervical cancer cells were maintained in either Eagle's Minimum Essential Medium (EMEM; SiHa and HeLa) or Dulbecco’s Modified Eagle’s Medium (MEM; DoTc2) supplemented with 10% Fetal Bovine Serum (FBS; ATCC). Ect1/E6E7 cells were maintained in Keratinocyte-Serum Free medium (GIBCO) with 0.1 ng/ml human recombinant EGF, 0.05 mg/ml bovine pituitary extract, and additional calcium chloride 44.1 mg/L. The ATCC authenticates all cell lines by examining their short tandem repeat (STR) DNA profiles. Cells were kept at 37°C in high humidity with an atmosphere of 95% air and 5% CO_2_. Cells were harvested using Trypsin-EDTA (0.25% Trypsin, 0.53 mM EDTA; ATCC) and counted using Trypan Blue 0.4% Solution (AMRESCO, Solon, OH) and hemacytometer counting chamber. For some experiments, cisplatin (1 mM; Sigma, St. Louis, MO) was reconstituted in distilled water and stored at -20°C until use. Cells were exposed to cisplatin at a concentration of 10 μM, 50 μM, and 100 μM in growth media for 4 h in culture media. Cisplatin-containing culture media was removed and replaced with fresh growth media 4 h later and cells incubated at 37°C overnight. For experiments investigating *PVT1* expression following hypoxia and immune response stimulation, SiHa cells were respectively treated with cobalt chloride (150 μM; Sigma) or interferon alpha (IFN-α; 10 μM; Sigma) for 48 h prior to RNA extraction (methods below). Finally, other SiHa cells were treated with cyclohexamide (10 μM; Sigma) for different time points before being subjected to lysis for Myc Western blotting.

### Transfections

Cells (2.0 x 10^5^) cells were plated into a 6-well dish and allowed to adhere overnight. Cells were then transfected with 2 small interfering RNA (siRNA) designed against *PVT1* combined in an equimolar ratio (20 or 40 nM; FlexiTube Hs_PVT1_5 and Hs_PVT1_6; Qiagen, Valencia, CA) or a negative control siRNA (AllStar Negative Control; Qiagen) conjugated with Alexa Fluor 488. Transfections were carried out using DharmaFECT1 (GE Dharmacon, Lafayette, CO) per the manufacturer’s instructions. Transfection efficiency was monitored via fluorescent microscopy and confirmed via quantitative PCR (qPCR) 48 h after transfection. siRNA transfections were performed in both SiHa and HeLa cervical cell lines and utilized in proliferation, apoptosis and cell death, and migration and invasion assays.

For locked nucleic acid (LNA) oligonucleotide transfections, cells (2.0 x 10^5^) were plated into a 6-well dish and the following day transfected with 10 nM PVT1_10 or PVT1_15 LNA (Exiqon, Woburn, MA) or a scrambled control LNA (Negative Control A, Exiqon) using DharmaFECT 1 (GE Dharmacon) at a concentration of 0.2 μl per 100 μl growth medium. The sequences of PVT1_10 LNA, PVT1_15 LNA and Negative Control A are 5'-AACACGTCTATACGC-3', 5’-AGATCACTGTAAATCC-3’ and 5'-GGCAGGATCTATGGCA-3', respectively. Transfection media was replaced with fresh growth media 24 h following transfection and downstream assays performed 48 h later. LNA-transfected SiHa cells were used to confirm the effects of siRNA transfection on cell proliferation, for determining the effects of *PVT1* knockdown on cisplatin responsiveness, Myc degradation experiments, and effects on nucleolin-dependent mRNA expression.

### RNA isolation, cDNA synthesis, and quantitative PCR (qPCR)

RNA was isolated using mirVana miRNA Isolation Kit (Ambion, Austin, TX) and cDNA synthesized using High-Capacity RNA-to-cDNA Kit (Applied Biosystems, Foster City, CA). For some experiments, live cultured cells were washed with 1x PBS and separated into cytoplasmic and nuclear RNA portions by use of nonionic detergents following the PARIS kit instructions (Life Technologies). RNA was isolated from each portion and DNase treated using DNA-free Kit (Life Technologies). Expression of *PVT1*, *MYC* and control *RPS18* was assessed using EvaGreen qPCR Mastermix (MidSci, St. Louis, MO) on a StepOnePlus Real-Time PCR System (Life Technologies). The thermal cycling program included an initial enzyme activation step at 95°C for 10min, followed by 40 cycles consisting of a 10sec denaturing step at 95°C, and a 1min annealing/extension step at 60°C for all primers. Fluorescent intensity was measured at 62°C at the end of each cycle. Forward and reverse primers for *PVT1* were 5’-CCGACTCTTCCTGGTGAAGC-3’ and 5’-GTATGGTCAGCTCAAGCCCA-3’, 5’-TACCCTCTCAACGACAGAG-3’ and 5’- TCTTGACATTCTCCTCGGTG-3’ for *MYC*, and 5’-CTTCAGTCGCTCCAGGTCTT-3’ for *RPS18* (for data normalization).s for *PVT1* were 5’-CCGACTCTTCCTGGTGAAGC-3’ and 5’-GTATGGTCAGCTCAAGCCCA-3’, 5’-TACCCTCTCAACGACAGAG-3’ and 5’- TCTTGACATTCTCCTCGGTG-3’ for *MYC*, and 5’-CTTCAGTCGCTCCAGGTCTT-3’ for *RPS18* (for data normalization).

Expression of miRNAs was conducted using TaqMan® MicroRNA assays (Life Technologies) for hsa-miR-1204 (002872), hsa-miR-1205 (002778), hsa-miR-1206 (002878), hsa-miR-1207-3P (002826), hsa-miR-1207-5P (241060_mat), hsa-miR-1208 (002880), and RNU48 (001006; for data normalization) according to manufacturer’s instructions. Briefly, RNA was isolated using mirVana miRNA Isolation Kit and cDNA synthesized using the TaqMan MicroRNA Reverse Transcription Kit (Life Technologies) with provided primers for each miRNA. qPCR amplification was then performed on a StepOnePlus Real-Time PCR System using the manufacturer’s parameters. All samples were analyzed in triplicate using 2− ΔΔCt method for relative gene expression, expressed as 2−ΔΔCt.

### RNA fluorescent in situ hybridization (FISH) and immunocytochemistry

ViewRNA TYPE 6 probe sets (Affymetrix, Santa Clara, CA) designed against *PVT1* (NR_003367.2) were used to visualize *PVT1* expression *in vitro*. Experiments were conducted according to manufacturer’s protocol using reagents provided in the QuantiGene ViewRNA ISH Cell Assay Kit (Affymetrix). Briefly, SiHa cells were seeded onto 12 mm circular coverslips and grown to 70–90% confluence before being fixed with 4% formaldehyde. Cells were then washed with 1X PBS and incubated for 5 min in Detergent Solution for permeabilization. Cells were washed once more and incubated with Working Protease Solution for 25 min. Probes were diluted (1:100) in pre-warmed Probe Set Diluent and incubated with the cells for 3 h at 40°C. Cells were washed, hybridized with Working PreAmplifier Mix Solution for 30 min at 40°C, washed again, and hybridized with Working Amplifier Mix Solution for 30 min at 40°C. After more washing, cells were hybridized with the labeled probes for 30 min at 40°C. Following this incubation, coverslips were washed, stained with DAPI, and mounted on glass slides. Cells were imaged with a Zeiss LSM510 confocal microscope located in the Children’s Research Institute Imaging Core at the Medical College of Wisconsin. Images were processed in Adobe Photoshop CS5.1.

For co-staining of Nucleolin with *PVT1* RNA FISH, immunocytochemistry was performed prior to the staining with DAPI step above. Cells were washed 3 times with PBS +1% BSA and 0.1% Tween-20 and then blocked at room temperature for 2 h in blocking buffer (PBS + 10% BSA, 0% normal donkey serum and 0.1% Tween-20). Coverslips were then incubated at 4°C overnight in blocking buffer containing a polyclonal antibody directed against Nucleolin (1:1000; Cell Signaling Technology, Danvers, MA). Cell were again washed 3 times and incubated for 1 h at room temperature in block solution containing a FITC-conjugated secondary antibody for visualization of Nucleolin. After a final wash, coverslips were mounted using Vectashield Antifade Mounting Medium with DAPI (Vector Laboratories, Burlingame, CA) and imaged as described above.

### Cell proliferation assay

Cell proliferation was measured using the Cell Proliferation ELISA BrdU (colorimetric) kit (Hoffmann-La Roche, Nutley, NJ) according to the manufacturer’s protocol. Briefly, 48 h after transfection, 1.0 x 10^4^ cells were plated in a 96-well dish and allowed to adhere overnight. Cells were then labeled with BrdU for 2 h, fixed, and incubated with anti-BrdU-POD. Unbound peroxidase conjugates were removed by washing. Substrate was then added and absorbance values (370 nM–492 nM) were measured 15 min later.

The PrestoBlue® Cell Viability assay was also used according to manufacturer’s instructions. SiHa cells were transfected with LNA as described above. Forty-eight h later, cells were plated onto a 96-well plate (1.0 x 10^4^ cells/well) and exposed to cisplatin (Sigma) at a range of concentrations between 10–200 μM for 4 h. Cisplatin culture media was removed and replaced with growth media after the 4 h exposure and then incubated at 37°C overnight. The following day, fluorescence values were measured after incubation of cells with the PrestoBlue® reagent for 1 h at 37°C.

### Cell death and apoptosis assays

Cell death was assayed using the Cell Death Detection ELISA Kit (Hoffmann-La Roche) as per manufacture directions. Briefly, 48 h after transfection, 0.5 x 10^5^ cells were harvested and lysed. Cellular nucleosomes were bound to the prepared ELISA plate through histone components. Anti-DNA-peroxidase was added, and unbound peroxidase conjugates were removed by wash. Substrate was then added and absorbance values were measured 15 min later (405 nM–490 nM). Apoptosis was characterized by measuring active caspase-3 (ng/mg of total cell lysate) using the Human Caspase-3 (Active) ELISA Kit (Life Technologies, Grand Island, NY). Seventy-two h after transfection, cells (1.0 x 10^6^) were lysed in Cell Extraction Buffer (Life Technologies) supplemented with Protease Inhibitor Cocktail (Sigma) and phenylmethanesulfonyl fluoride (Sigma).

### Cell migration and invasion assays

Forty-eight h after transfection, media containing serum was removed and cells were washed with 1x PBS then cultured for 24 h in serum-free EMEM. After the starvation period, cells were harvested using HyQTase and plated (1 x 10^5^ cells) and onto Corning Transwell Permeable Supports (Corning, Tewksbury, MA). For migration assays, Transwell membranes were equalized in growth media 24 h before cell seeding. For invasion assays, Transwell membranes were coated with Matrigel Matrix (Corning; 1:6 in EMEM) and dried for 6 h at 37°C. Cells were cultured in the presence and absence of chemoattractant (EMEM + 20% FBS) and harvested 6 h or 48 h later for analysis of migration and invasion, respectively. Non-migrant cells were removed from the upper face of the Transwell membrane with a cotton swab, while migrant cells were fixed and stained with Crystal Violet (EMD Millipore, Billerica, MA) and visualized/counted using light microscopy.

### Western blotting

Fresh frozen cervical tissue or 3.6 x 10^6^ cells were washed with 1x PBS and lysed for 10 min on ice using Cell Extraction Buffer (Life Technologies) supplemented with 1 mM PMSF (Sigma) and a Protease Inhibitor Cocktail (Sigma). Undigested cellular debris was pelleted by centrifugation at 10,000 RPM and remaining lysate was quantified using the Bradford Assay (Sigma). Lysate (20 μg) was run out on a Criterion Tris-HCl polyacrylamide gel (Bio-Rad Laboratories, Hercules, CA), transferred to PVDF membranes, blocked with 1X TBS + 10% nonfat dry milk at room temperature for 1 h, and incubated with primary antibody (Myc or Nucleolin, 1:1000; Cell Signaling Technology) overnight at 4°C. The following day, membranes were washed, incubated for 1 h at room temperature with HRP-conjugated goat anti-rabbit IgG (1:5,000; Cell Signaling Technology), and developed using ECL (Life Technologies). Chemiluminescence was measured using Molecular Imager ChemiDoc XRS+ (Bio-Rad Laboratories) and visualized protein bands were quantified using Image Lab Software (Bio-Rad Laboratories).

### RNA affinity chromatography and mass spectrometry sequencing

Several overlapping sense and antisense single-stranded DNA oligonucleotides (ssDNA oligos; 100 nt long) were generated against the complete sequences of exons 2 and 9 of *PVT1* (NR_003367.3; Eurofins MWG Operon, Huntsville, AL). Exons 2 and 9 were chosen based on their increased propensity for protein binding as determined *in silico*. ssDNA were hybridized to obtain double-stranded DNA (dsDNA), all of which had a T7 promoter sequence at the 5’end. The T7 promoter-containing dsDNA was *in vitro*-transcribed using the MaxiScript T7 kit (Ambion) and resultant RNA was then 3’-biotinylated with the Pierce Desthiobiotinylation kit (ThermoScientific). Desthiobiotinylated RNAs were incubated with streptavidin-bound magnetic beads from Pierce Magnetic RNA-Protein Pull-Down Kit (Thermo Scientific) as per the manufacturer’s protocol. Following washing, 200 μg of SiHa whole cell lysate was added to the RNA-bound streptavidin magnetic beads and incubated at 4°C with gentle agitation for 90 min. After 3 washes with Wash Buffer (Thermo Scientific 20164), the RNA bound proteins were eluted in SDS sample buffer and resolved by SDS-PAGE on a 12% acrylamide gel. Following silver staining, protein bands of interest were Mass Spectrometry sequenced at Taplin Mass Spectrometry Facility (Harvard Medical School, Boston, MA). Mass spectrometry “hits” were analyzed for the number of unique and total peptides obtained and area under the curve. Each hit was *in silico* trypsin digested and the number of peptides obtained with *in silico* digestion was compared with the number of peptides obtained in the mass spectrometry results. Only those proteins that had 20% or more of the trypsin-digestible fragments present were considered to be true hits. Western blotting (as described above) following RNA affinity chromatography was further used to validate true hits.

### RNA immunoprecipitation

RNA immunoprecipitation (RIP) assays were performed using the Magna RIP RNA-Binding Protein Immunoprecipitation Kit (EMD Millipore) following manufacturer’s instructions. Total cell lysate from 1.7 x 10^7^ SiHa cells was used for immunoprecipitation with Myc and Nucleolin antibodies (same as for Western blotting). As a nonspecific IgG control, purified Rabbit IgG was run in parallel. Following protein digestion, RNA was extracted and DNase treated using DNA-free Kit (Life Technologies). Levels of *PVT1* were quantified in input and immunoprecipitated RNA.

### Data Analysis and Statistics

Experiments were performed in triplicates and differences between mean data were analyzed using Student’s *t* tests (two-tailed). Results have been graphed as means ± standard error of the mean. Cisplatin dose-response curves were generated using a variable slope model. All statistical analyses and graphing of data were performed using GraphPad Prism 6.0 (La Jolla, CA), except for survival analysis which was performed using SPSS11.0 software (Chicago, IL). Kaplan-Meier survival curves were plotted and compared using log-rank (Mantel-Cox) tests. P values less than 0.05 were considered significant.

## Results

### *PVT1* expression is upregulated in tumors from cervical cancer patients and correlated with poorer survival

**Fig 1:**
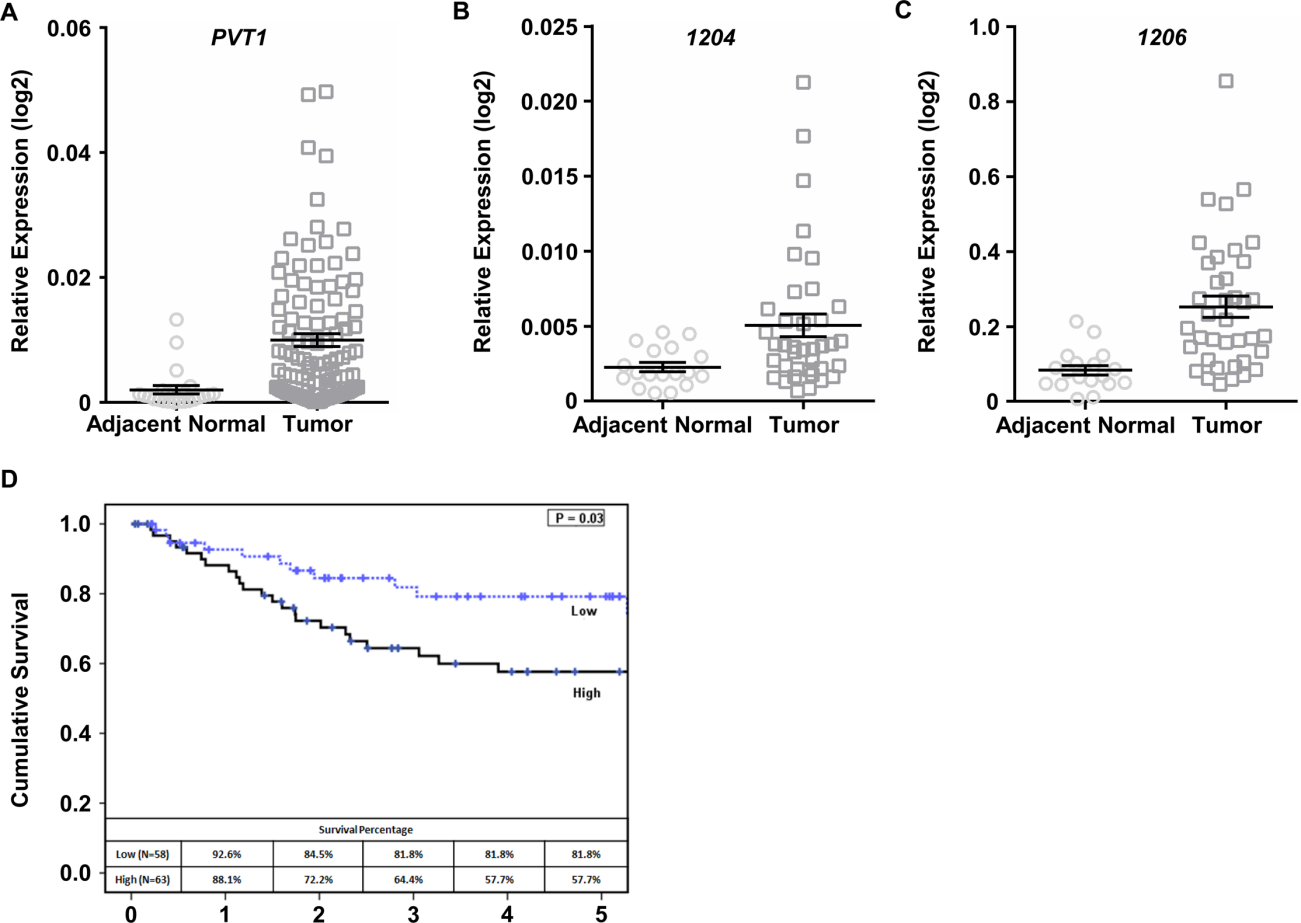
*PVT1* and local miRNA expression in cervical cancer tissue. (A) *PVT1* expression was significantly higher in primary cervical tumors (n = 127) compared to adjacent normal cervical tissue (n = 30; p<0.001). Expression of miRNAs 1204 (B; p<0.05) and 1206 (C; p<0.001), but not other local miRNAs, was also significantly higher in cervical tumor tissue (n = 38) compared to adjacent normal tissue (n = 18). (D) Kaplan-Meier plots revealed an association of higher tumor *PVT1* levels with significantly poorer survival times compared to lower *PVT1* levels (p = 0.03).

*PVT1* is a multi-exon gene with its contiguous genomic region containing a cluster of 6 annotated microRNAs (miRNAs). *PVT1* and local miRNA expression levels were determined by qPCR for cancerous and adjacent normal tissues from cervical cancer patients. *PVT1* expression was significantly higher in tumors from cervical cancer patients versus adjacent normal tissue (n = 127 tumor; n = 30 adjacent normal; p<0.001; Fig 1A). Expression of miRNAs 1204 and 1206 (but not 1205, 1207-3p, 1207-5p, or 1208; S1A–S1D Fig) was significantly higher in tumor versus normal adjacent cervical tissue (Fig 1B and 1C). Of note, because *PVT1* and *MYC* are frequently co-amplified in cancer, we additionally examined expression of *MYC* in cervical cancer and adjacent normal tissues. Although we observed a trend toward increased *MYC* in cancer compared to adjacent normal, the difference in average expression of *MYC* was not statistically significant between these two groups (S1E Fig).

The 127 cervical cancer patients were divided into high and low *PVT1*-expressing groups, using the median *PVT1* expression level, to investigate their correlation with prognosis. Using Kaplan-Meier survival analysis and log-rank tests, we found that high *PVT1* expression was significantly correlated with shorter cumulative survival time for cervical cancer patients (p = 0.03; Fig 1D). Together, these data suggest that, in general, *PVT1* expression is elevated in cervical cancer tumors and that the higher the expression of *PVT1*, the poorer the prognosis.

### *PVT1* expression in cervical cancer cell lines

**Fig 2:**
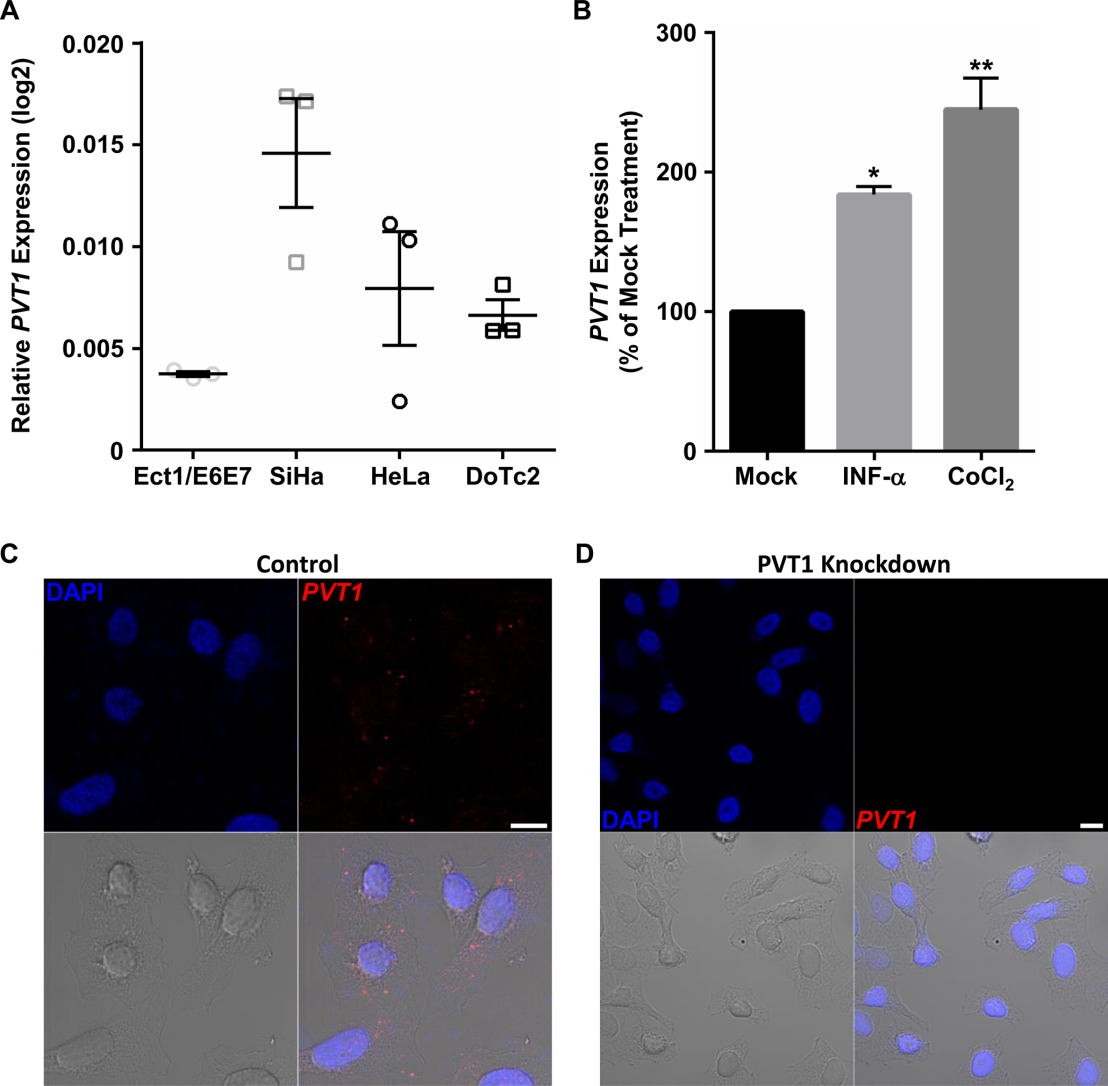
*PVT1* expression in cervical cancer cells. (A) *PVT1* expression in commercially available cervical cell lines. Lowest *PVT1* expression was observed in HPV 16 E6/E7-transformed cells derived from normal ectocervix (E6/E7-Ecto), while SiHa cervical cancer cells displayed the highest *PVT1* expression compared to 2 other cervical cancer-derived lines (HeLa and DoTc2). (B) Expression of *PVT1* in SiHa cells was further increased upon 48h treatment with INF-α (10 μM) or the hypoxia mimetic cobalt chloride (CoCl_2_; 150 μM). Representative images of RNA FISH experiments in SiHa cells transfected with either control (C) or *PVT1* (D) LNAs. Control LNA-transfected cells exhibited punctate signals for *PVT1* (red) in both the nucleus (stained with DAPI in blue) and the cytoplasm. Both nuclear and cytoplasmic *PVT1* staining was absent in *PVT1* LNA-transfected cells. Phase image (bottom left panel) depicts cell morphology. Scale bar (white) = 10 μm. *p<0.05, **p<0.01.

To begin our *in vitro* examination into the role of *PVT1* in cervical cancer cells, we determined *PVT1* expression levels in various commercially available cervix-derived cell lines via qPCR (Fig 2A). Out of 4 cervical lines, HPV 16-positive SiHa cells exhibited the highest *PVT1* expression, while the lowest *PVT1* expression was observed in HPV 16 E6/E7-transformed cells derived from normal ectocervix (Ect1/E6E7). Next, we sought to identify specific oncogenic stressors that may drive enhanced *PVT1* expression in cervical cancer. To this end, we exposed SiHa cells (used for the remainder of our studies) to various transformative stimuli prior to measuring the effects on *PVT1* expression via qPCR. Interestingly, we found that *PVT1* expression in SiHa cells was significantly increased in response to 48 h incubation with INF-α or the hypoxia mimetic, cobalt chloride (CoCl_2_; Fig 2B). Other stimuli, such as epidermal growth factor (EGF), insulin-like growth factor (IGF), phorbol 12-myristate 13-acetate (protein kinase C activator), and gamma-irradiation did not significantly affect *PVT1* expression (data not shown). Finally, we investigated subcellular localization of *PVT1* using RNA FISH and found expression of *PVT1* in both the cytoplasm and nucleus of SiHa cells. Further, control antisense-transfected SiHa (Fig 2C) exhibited similar nuclear and cytoplasmic *PVT1* staining, of which was absent in *PVT1* knockdown cells (Fig 2D). In summary, *PVT1* is expressed in various cell lines derived from human cervix, can be augmented by immune and hypoxic stimuli, and is localized throughout the nucleus and cytoplasm suggesting that this lncRNA may participate in various cervical cancer cell processes, possibly via more than one unique mechanism.

### *PVT1* silencing decreases cervical cancer cell proliferation, migration and invasion

**Fig 3:**
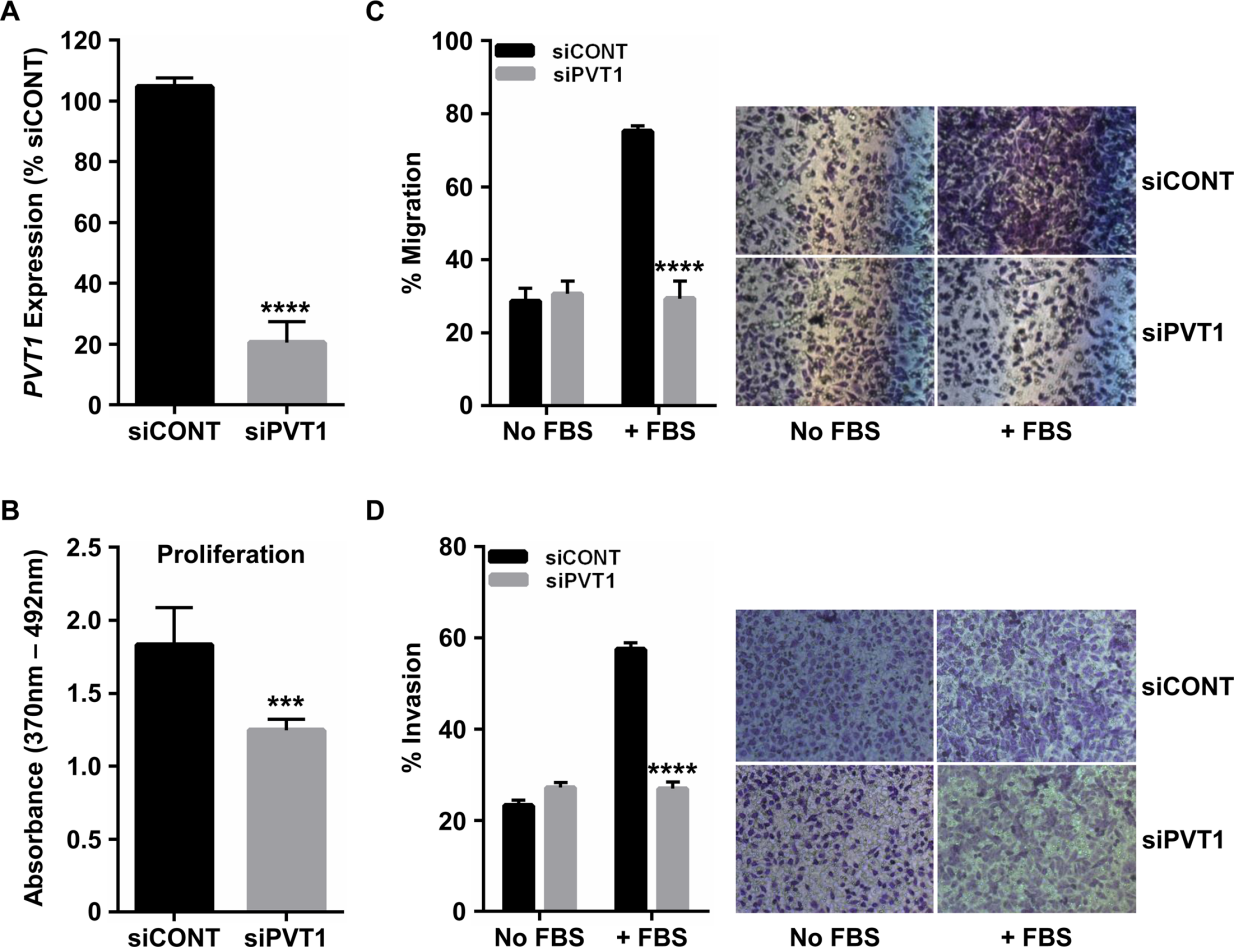
*PVT1* promotes cervical cancer cell proliferation, migration and invasion. (A) Transfection of SiHa cervical cancer cells with siRNAs targeting *PVT1* (siPVT1) resulted in an approximate 70% knockdown in *PVT1* lncRNA expression as compared to cells transfected with a scrambled control siRNA (siCONT). (B) SiHa cells transfected with siPVT1 exhibited a significant decrease in proliferation compared to siCONT cells. Transfected SiHa cells were also assessed for changes in (C) migration and (D) invasion 6 h or 48 h following introduction of chemoattractant (FBS), respectively. siPVT1 cells showed a significant decrease in both cell migration and invasion compared to siCONT cells. Quantitative results are graphed on the left, while representative images are on the right. ***p<0.001, ****p<0.0001

Next, SiHa cells transfected with *PVT1*-targeted siRNA (siPVT1) were utilized to examine the effects of this lncRNA on cervical cancer cell proliferation and motility. Transfection with *PVT1*-targeting siRNAs resulted in, on average, 70% knockdown of *PVT1* compared to control-transfected cells (Fig 3A). Further, there was no significant difference in local miRNA (1204–1208) or *MYC* mRNA expression in siPVT1 versus scrambled control-transfected cells (siCONT; S2 Fig). siPVT1-transfected SiHa cells exhibited a significant decrease in BrdU incorporation, a measure of replicating cells, at 72 h post-transfection compared to siCONT, suggesting that *PVT1* plays a role in driving cell proliferation (Fig 3B). These effects of *PVT1* knockdown on SiHa proliferation were also confirmed by transfection with *PVT1*-targeted LNA. Finally, in comparison to siCONT cells, siPVT1 cells displayed a significant decrease in migration (Fig 3C) and invasion (Fig 3D) potential. Of note, these effects of *PVT1* knockdown are mimicked in another cervical cancer cell line, HeLa (S3 Fig), providing further support for a role of this lncRNA in cervical carcinogenesis.

### *PVT1* silencing increases apoptosis and response to cisplatin in cervical cancer cells

**Fig 4:**
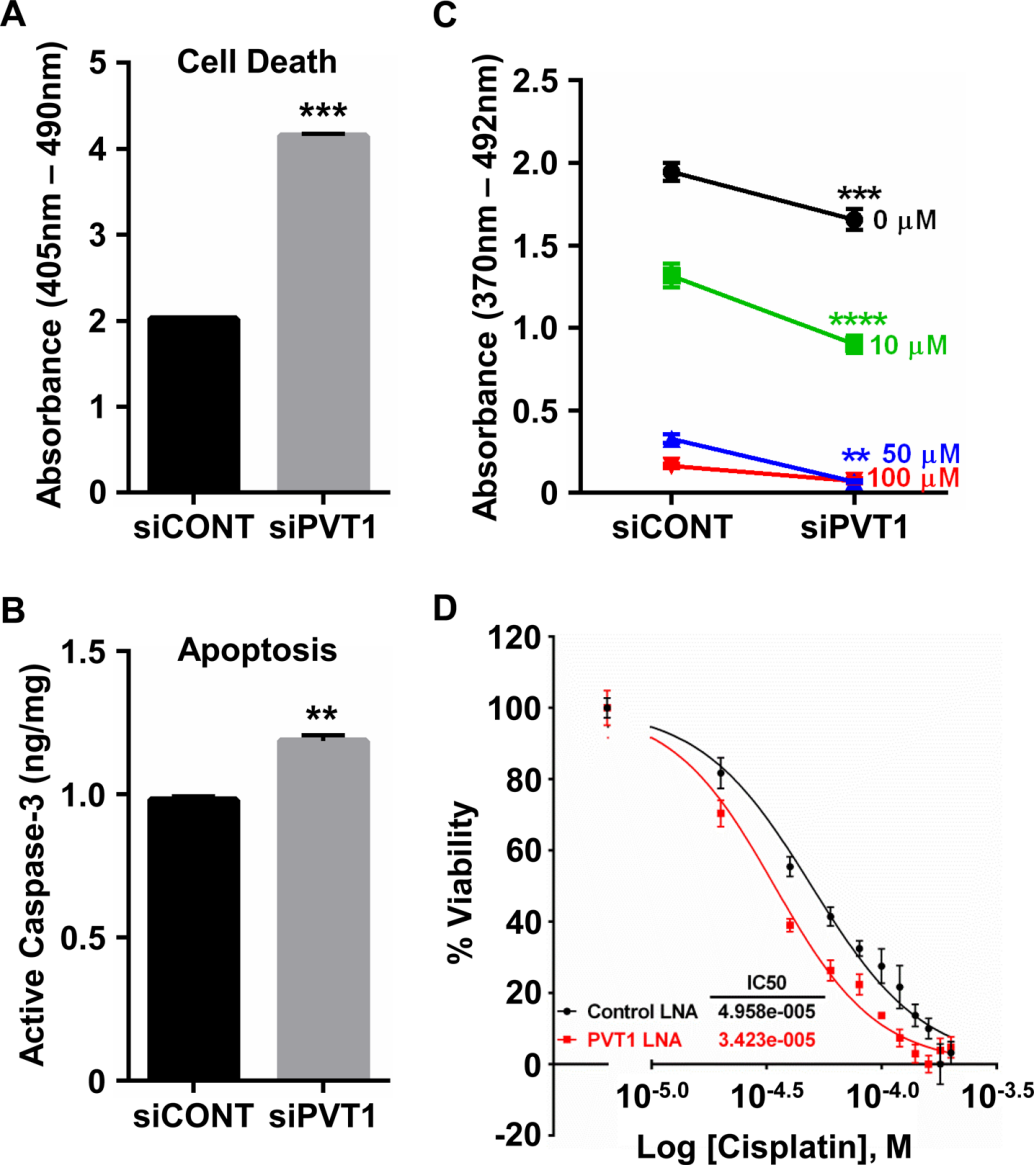
*PVT1* inhibits cervical cancer cell death and cisplatin sensitivity. siPVT1 cells exhibited a significant increase in cell death (A) and apoptosis (B) compared to siCONT cells. (C) Proliferation of siPVT1 SiHa cells was significantly decreased in response to multiple doses of cisplatin. (D) Dose-response curve for control and *PVT1* LNA-transfected SiHa cells following 4 h treatment with cisplatin. The IC_50_ value of the *PVT1* knockdown cells was significantly decreased compared to control knockdown cells (p<0.0001), suggesting a role for this lncRNA in cisplatin resistance. **p<0.01, ***p<0.001, ****p<0.0001

To date, siRNA-mediated knockdown strategies targeting *PVT1* have demonstrated its role in cisplatin sensitivity in malignant pleural mesothelioma [25] and gastric cancer cells [26]. However, the anti-apoptotic signature induced by *PVT1* in other cancer cell types [8,18,20,26] may indicate that its contribution to cisplatin resistance is more far-reaching. Thus, our next experiments were designed to investigate the role of *PVT1* in apoptosis and cisplatin sensitivity in cervical cancer cells. Extending the previously mentioned findings, our results show that *PVT1* knockdown in SiHa cells significantly increased levels of cytoplasmic histone-associated DNA fragments (Fig 4A) and active caspase-3 (Fig 4B), suggesting that *PVT1* can also contribute to cervical carcinogenesis via inhibition of cell death and apoptosis. Further, *PVT1* knockdown by either siRNA (Fig 4C) or LNA oligonucleotides (Fig 4D) leads to increased responsiveness of SiHa cells to cisplatin. Collectively, the cell phenotypic (loss of *PVT1*) functional data suggests that *PVT1* is required for proliferation, maintenance and cisplatin resistance of cervical cancer cells.

### *PVT1* interacts with Myc and Nucleolin proteins in cervical cancer

Many lncRNAs exert their functions via binding specific protein partners. *PVT1* has been shown to bind and stabilize the Myc protein by preventing its phosphorylation at threonine 58 to promote carcinogenesis in breast cancer cells [19]. However, although RNA immunoprecipitation results suggest that Myc and *PVT1* associate in SiHa cells (S4A Fig), western blotting of protein lysate from cells exposed to siRNA-mediated knockdown of *PVT1* did not reveal significant differences in total or T58-phosphorylated Myc levels (S4B Fig), nor in the rate of Myc degradation (S4C and S4D Fig). Thus, it appears that *PVT1* likely exerts its effects through an alternative mechanism in cervical cancer cells.

**Fig 5:**
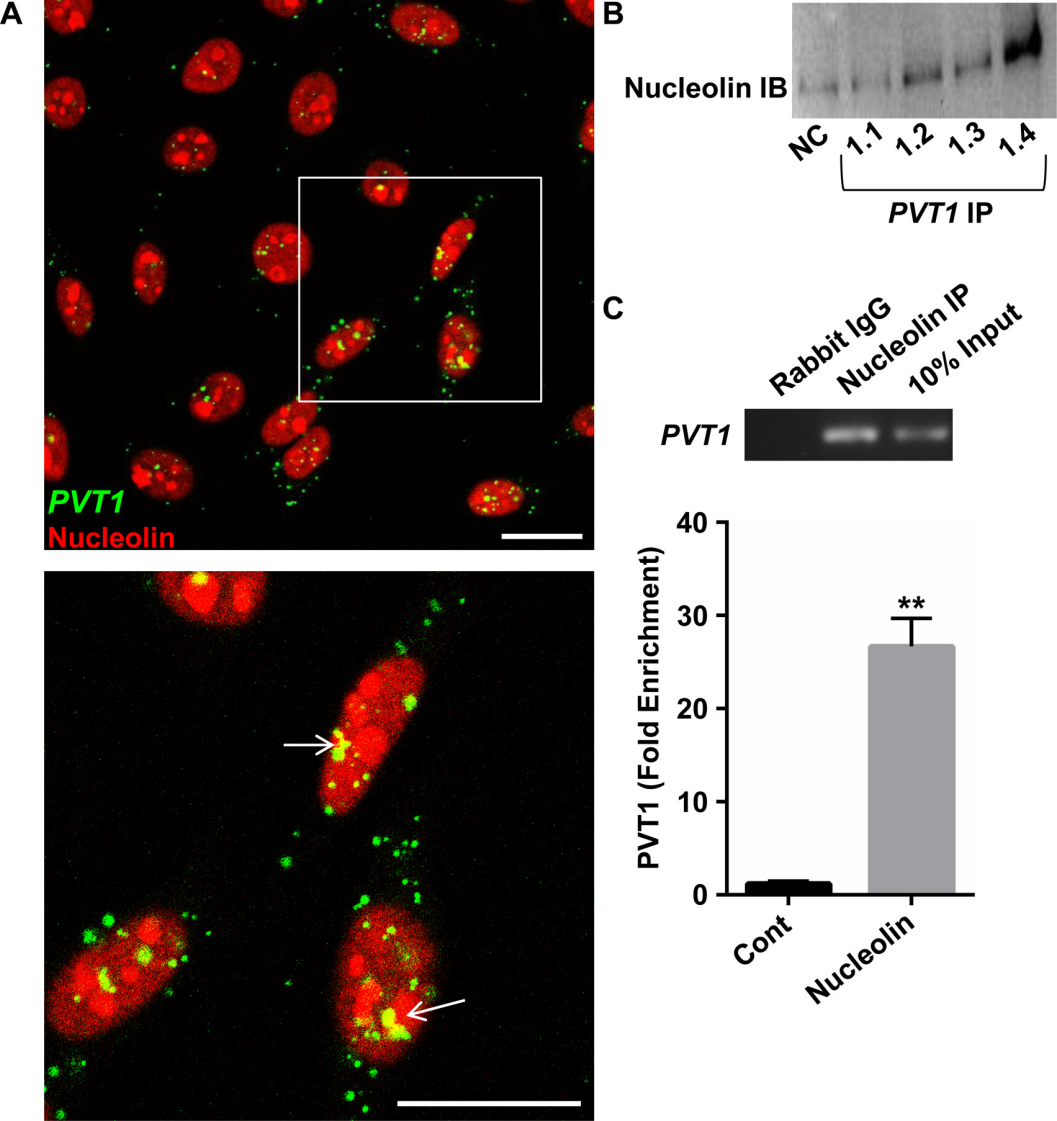
*PVT1* interacts with nucleolin in SiHa cells. (A) SiHa cells stained for *PVT1* (green) and nucleolin (red), and DAPI. Boxed cells (top panel) are at higher magnification (bottom panel). White arrows show *PVT1*-nucleolin colocalization. Scale bars = 20μM. (B) Western blot for Nucleolin of SiHa protein captured by *PVT1* RNA affinity chromatography. (C) *PVT1* RNA was immunoprecipitated with anti-Nucleolin, but not the negative control rabbit IgG (Cont). **p<0.01

To this end, using RNA affinity chromatography in conjunction with mass spectrometry sequencing, we identified potential protein binding partners of *PVT1 in vitro* using whole-cell lysates from SiHa cervical cancer cells. Of the several proteins identified, we chose to further examine the multifaceted DNA and RNA binding protein, Nucleolin, as it could account for the wide-reaching effects of *PVT1* knockdown. We confirmed the interaction of *PVT1* and Nucleolin in SiHa cells via their colocalization using combined RNA FISH and immunocytochemistry (Fig 5A). Next, we performed reciprocal capture of Nucleolin or *PVT1* with complementary approaches. In the first approach, we identified Nucleolin by western blotting with anti-Nucleolin when *PVT1* RNA was captured by RNA affinity chromatography (Fig 5B). In the second approach, significant *PVT1* enrichment was observed in anti-Nucleolin (Fig 5C) immunoprecipitates. Because *PVT1* has previously been shown to bind to specific proteins and mediate their stability [17,19], we next investigated the effect of *PVT1* knockdown on Nucleolin protein expression. Somewhat surprisingly, LNA-mediated knockdown of *PVT1* had no significant effect on total Nucleolin protein levels (S5 Fig), suggesting a disparate function of *PVT1*-Nucleolin binding. Work is ongoing to elucidate the mechanistic properties of the *PVT1*-Nucleolin interaction in cervical cancer cells.

## Discussion

The current study revealed that *PVT1* expression levels in cervical cancer tumors were significantly higher than in adjacent noncancerous tissue and that high *PVT1* expression levels correlated with patients’ poorer overall survival. *PVT1* expression was also examined *in vitro*, with the SiHa cervical cancer cell line expressing the highest levels of the lncRNA compared to others. Moreover, *PVT1* expression in SiHa cells was further augmented under hypoxic conditions or following immune response stimulation by INF-α. Functionally, *PVT1* knockdown in cervical cancer cells was associated with decreased proliferation, migration and invasion and increased apoptosis and cisplatin sensitivity, suggesting that this lncRNA likely plays a pivotal and multifaceted role in cervical carcinogenesis. Finally, the effects of *PVT1* on cervical cancer cells may be facilitated by its association with the protein Nucleolin.

The human *PVT1* gene is a long intergenic noncoding RNA that gives rise to multiple splice variants. Ensembl data suggests the presence of at least 25 unique human *PVT1* transcripts [27] and exon 2 of *PVT1* can also form a circular RNA (circRNA) that is highly expressed in HeLa cervical cancer cells [28], thus making the study of *PVT1* function particularly challenging. Here, we have used siRNA and LNA-mediated knockdown of *PVT1* to study its role in cervical carcinogenesis. While both methods of knockdown were particularly efficient in knocking down multiple regions along the full-length *PVT1* transcript (with exception of primers designed to span exons 1 and 2), only LNA-transfected cells were effective in knocking down the exon 2-derived circRNA (S1 Table). Further, our RNA FISH images (Fig 2C and 2D) clearly demonstrate that knockdown of *PVT1* results in complete loss of staining for both nuclear and cytoplasmic *PVT1*. Due to the fact that the probes used for this assay cover 8 (NR_003367.2) out of 9 (NR_003367.3) exons of *PVT1* and that the signal from each individual probe can be amplified up to 96,000-fold, we conclude that most, if not all, *PVT1* isoforms are being affected by our knockdown. Thus, while the current manuscript indicates that *PVT1* plays a pivotal role in cervical carcinogenesis, future experiments are required to definitively pinpoint the precise splice variant(s) responsible for the oncogenic effects of *PVT1* in cervical cancer.

The *8q24* region of the human genome, which contains *MYC* and *PVT1*, is one of the most common sites of cancer-related amplifications [29,30]. The *MYC* oncogene encodes a well-characterized transcription factor that plays a role in various cancer-related processes such as cell cycle progression, cellular transformation, and apoptosis [31] making it essential that studies investigating *PVT1* and its cellular mechanisms include concurrent examination of *MYC*. Co-amplification and expression of *MYC* and *PVT1* has been shown to play a role in the promotion of the malignant phenotype in malignant pleural mesothelioma [25], ovarian and breast cancer [8,19], and neuroblastoma [32]. Here, we show that *PVT1* expression is significantly higher in cervical cancer tissue compared to normal controls (Fig 1A). Unlike in other cancers, however, we did not observe a concurrent increase in *MYC* mRNA levels in cervical tumors (S1E Fig). Further, using publicly available TCGA data from cBioPortal [33,34], we found that 13% (40 cases) of cervical cancer tumors have amplification and/or overexpression of *PVT1*, yet co-amplification of *PVT1* and *MYC* only occurs in 27.5% (11/40 cases) and *PVT1* amplification or mRNA upregulation with no alteration in *MYC* occurs in 65% (26/40 cases). Although we also found evidence of physical interaction between *PVT1* and Myc protein in SiHa cells (S4A Fig), we did not observe any significant effects of *PVT1* knockdown on Myc protein levels or stabilization (S4B and S4C Fig). Collectively, these data suggest that co-regulation of *PVT1* and *MYC* expression may not be as common in cervical cancer as it is in other cancer types and that the result of their interaction is unlike that described previously [19]. Future work is necessary to elucidate the biological function of the *PVT1*-Myc association in cervical cancer.

We have provided three lines of evidence confirming an interaction between *PVT1* and Nucleolin in SiHa cells (Fig 5). Nucleolin is a multifunctional protein that is well known to be dysregulated in disease [35,36]. Although abundantly expressed in the nucleolus, Nucleolin also localizes to cytoplasmic and plasma membrane compartments [37–39], and its subcellular localization can indicate its functional role in different cell types [35]. Our RNA FISH/immunocytochemistry data suggest that, in SiHa cells, colocalization of Nucleolin and *PVT1* is chiefly nuclear/nucleolar (Fig 5A). Nucleolin in these compartments regulates ribosomal biogenesis, facilitates rDNA transcription, and governs oncogene expression [35]. Interestingly, these regulatory functions of Nucleolin are intimately tied to cellular stress responses under conditions such as cancer-related hypoxia and immune activation via viral infection [40–43]; stimuli we demonstrate here to also significantly augment *PVT1* expression in SiHa cells (Fig 2B) and that are especially relevant in cervical disease [44–48]. Thus, our future research aims to examine the importance of the *PVT1*-Nucleolin interaction in the context of these processes, and whether they underlie the oncogenic effects of *PVT1* in cervical cancer *in vivo*.

In summary, we propose that high expression levels of *PVT1* contribute to the cervical cancer phenotype via modulation of cell proliferation, apoptosis, and cell motility. Perhaps most importantly, high *PVT1* expression correlates with poorer survival outcome in cervical cancer patients and may play a central role in resistance of cervical cancer cells to cisplatin. *In vitro*, *PVT1* expression is significantly augmented by cancer-related hypoxic and immune-stimulatory treatments, suggesting possible mechanisms underlying its heightened expression in cervical cancer. Finally, we have identified Nucleolin as a protein binding partner of *PVT1* in cervical cancer cells. Future work in our laboratory is aimed to fully elucidate the functional consequences of *PVT1*-Nucleolin binding as well as to determine what role, if any, the *PVT1*-Myc interaction plays in cervical carcinogenesis.

## Supporting Information

S1 Fig Local miRNA and MYC expression in cervical cancer. (A-D) miR1205, 1207, and 1208 expression was not significantly different between adjacent normal (n = 18) and cancer tissue (n = 38). (E) MYC mRNA levels were also not significantly different between the two groups. (F) *PVT1* expression in commercially available cervical cell lines. Lowest *PVT1* expression was observed in HPV 16 E6/E7-transformed cells derived from normal ectocervix (E6/E7-Ecto), while SiHa cervical cancer cells displayed the highest *PVT1* expression compared to 2 other cervical cancer-derived lines (HeLa and DoTc2).
(TIF)

S2 Fig siRNA-mediated *PVT1* knockdown did not affect expression of any local miRNAs nor MYC. (TIF)

S3 Fig *PVT1* promotes cervical cancer cell proliferation, migration and invasion in HeLa cells. (A) Transfection of HeLa cervical cancer cells with siRNAs targeting *PVT1* (siPVT1) resulted in an approximate 60% knockdown in *PVT1* lncRNA expression as compared to cells transfected with a scrambled control siRNA (siCONT). (B) HeLa cells transfected with siPVT1 exhibited a significant decrease in proliferation compared to siCONT cells. Transfected HeLa cells were also assessed for changes in (C) migration and (D) invasion 6 h or 48 h following introduction of chemoattractant (FBS), respectively. siPVT1 cells showed a significant decrease in both cell migration and invasion compared to siCONT cells. Quantitative results are graphed on the left, while representative images are on the right. **p<0.01, ***p<0.001, ****p<0.0001
(TIF)

S4 Fig PVT1 binds, but does not stabilize, Myc in SiHa cells. (A) PVT1 from SiHa total cell lysate immunoprecipitated with a MYC antibody, but not the negative control rabbit IgG (Cont). (B) PVT1 knockdown in SiHa cells did not significantly affect p58 phospho-MYC or total MYC protein. (C,D) Degradation of MYC protein was also not significantly affected by PVT1 knockdown. **p<0.01
(TIF)

S5 Fig *PVT1* knockdown does not affect Nucleolin protein levels. (TIF)

S1 Table Percent knockdown of different *PVT1* regions achieved with siRNA or LNA. * denotes primer set used throughout the manuscript.
(XLSX)
