# Supplementary figures and images for: The lncRNA PVT1 Contributes to the Cervical Cancer Phenotype and Associates with Poor Patient Prognosis
Source: PLoS One. 2016 May 27;11(5):e0156274. doi: 10.1371/journal.pone.0156274 (PMC4883781; doi:10.1371/journal.pone.0156274)

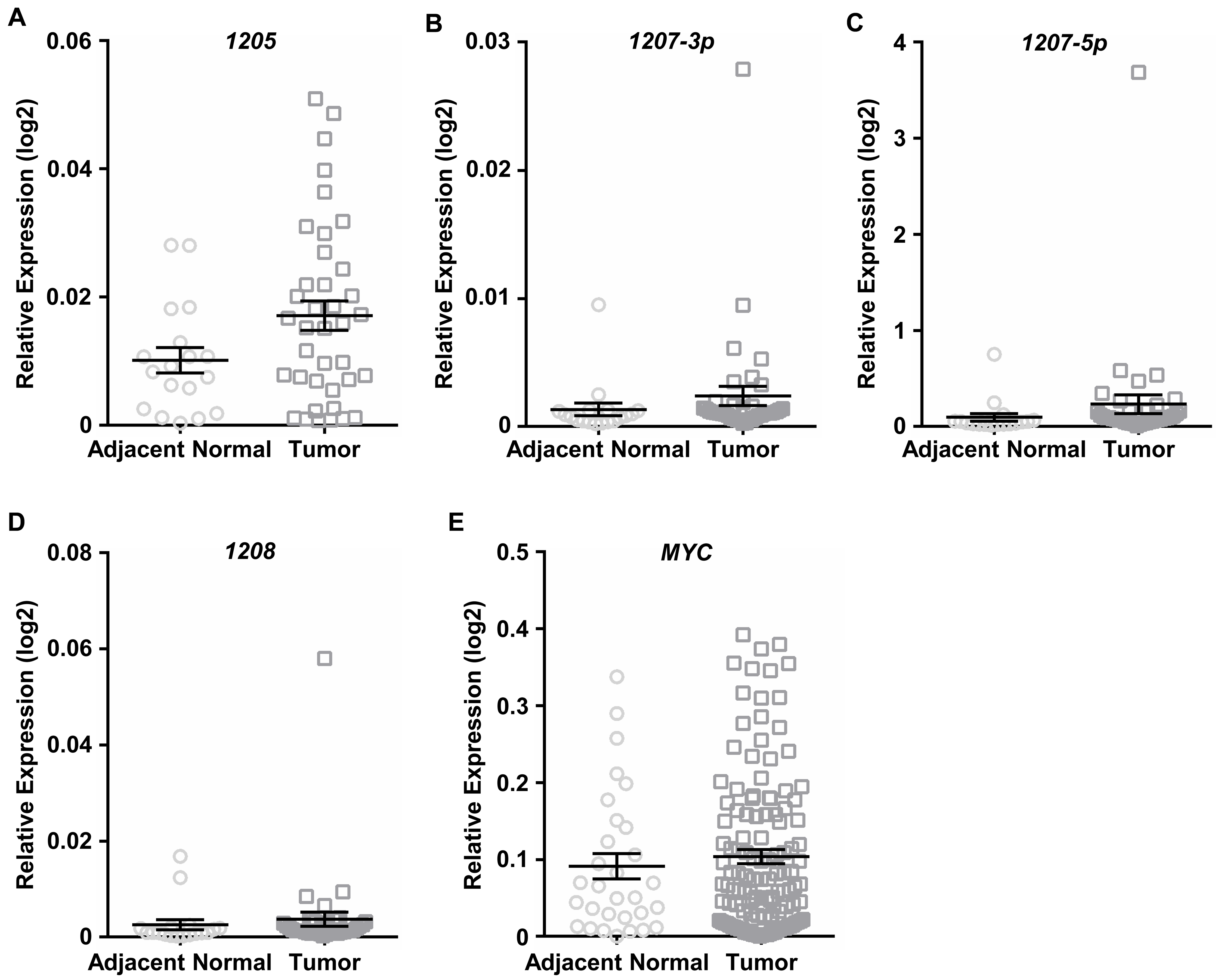

Supplement: S1 Fig — (A-D) miR1205, 1207, and 1208 expression was not significantly different between adjacent normal (n = 18) and cancer tissue (n = 38). (E) MYC mRNA levels were also not significantly different between the two groups. (F) PVT1 expression in commercially available cervical cell lines. Lowest PVT1 expression was observed in HPV 16 E6/E7-transformed cells derived from normal ectocervix (E6/E7-Ecto), while SiHa cervical cancer cells displayed the highest PVT1 expression compared to 2 other cervical cancer-derived lines (HeLa and DoTc2). (TIF) [file pone.0156274.s001.tif]

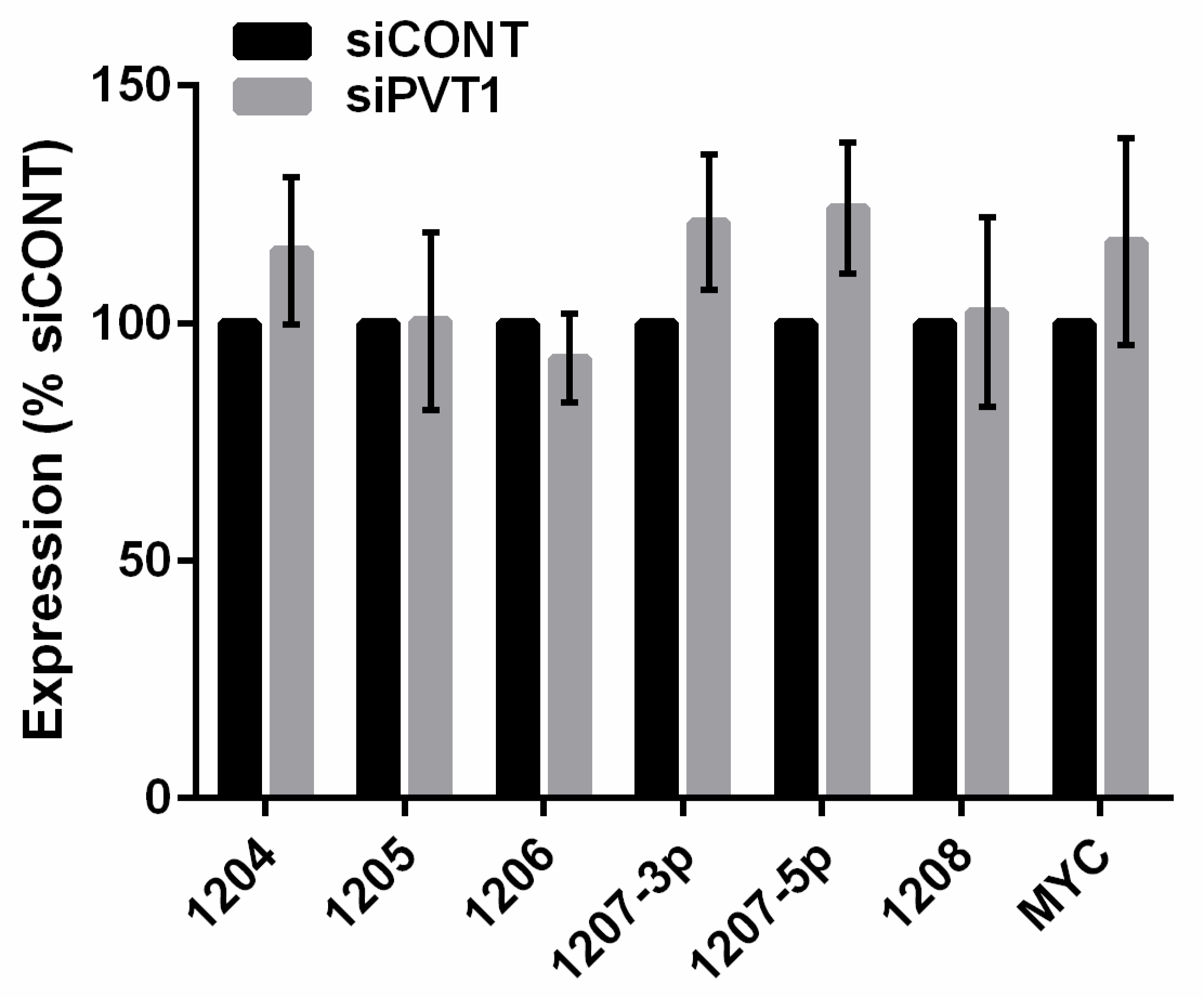

Supplement: S2 Fig — (TIF) [file pone.0156274.s002.tif]

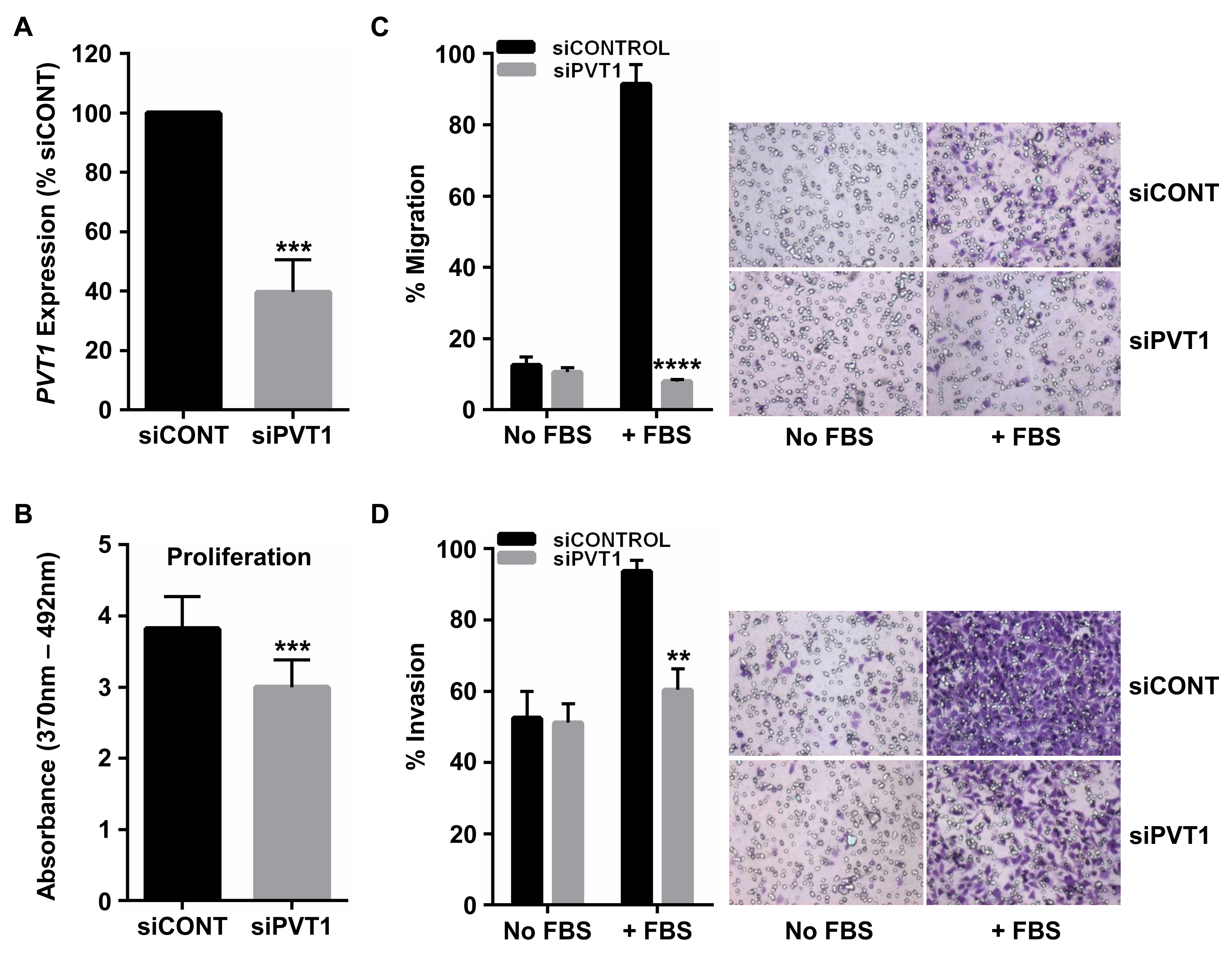

Supplement: S3 Fig — (A) Transfection of HeLa cervical cancer cells with siRNAs targeting PVT1 (siPVT1) resulted in an approximate 60% knockdown in PVT1 lncRNA expression as compared to cells transfected with a scrambled control siRNA (siCONT). (B) HeLa cells transfected with siPVT1 exhibited a significant decrease in proliferation compared to siCONT cells. Transfected HeLa cells were also assessed for changes in (C) migration and (D) invasion 6 h or 48 h following introduction of chemoattractant (FBS), respectively. siPVT1 cells showed a significant decrease in both cell migration and invasion compared to siCONT cells. Quantitative results are graphed on the left, while representative images are on the right. **p<0.01, ***p<0.001, ****p<0.0001 (TIF) [file pone.0156274.s003.tif]

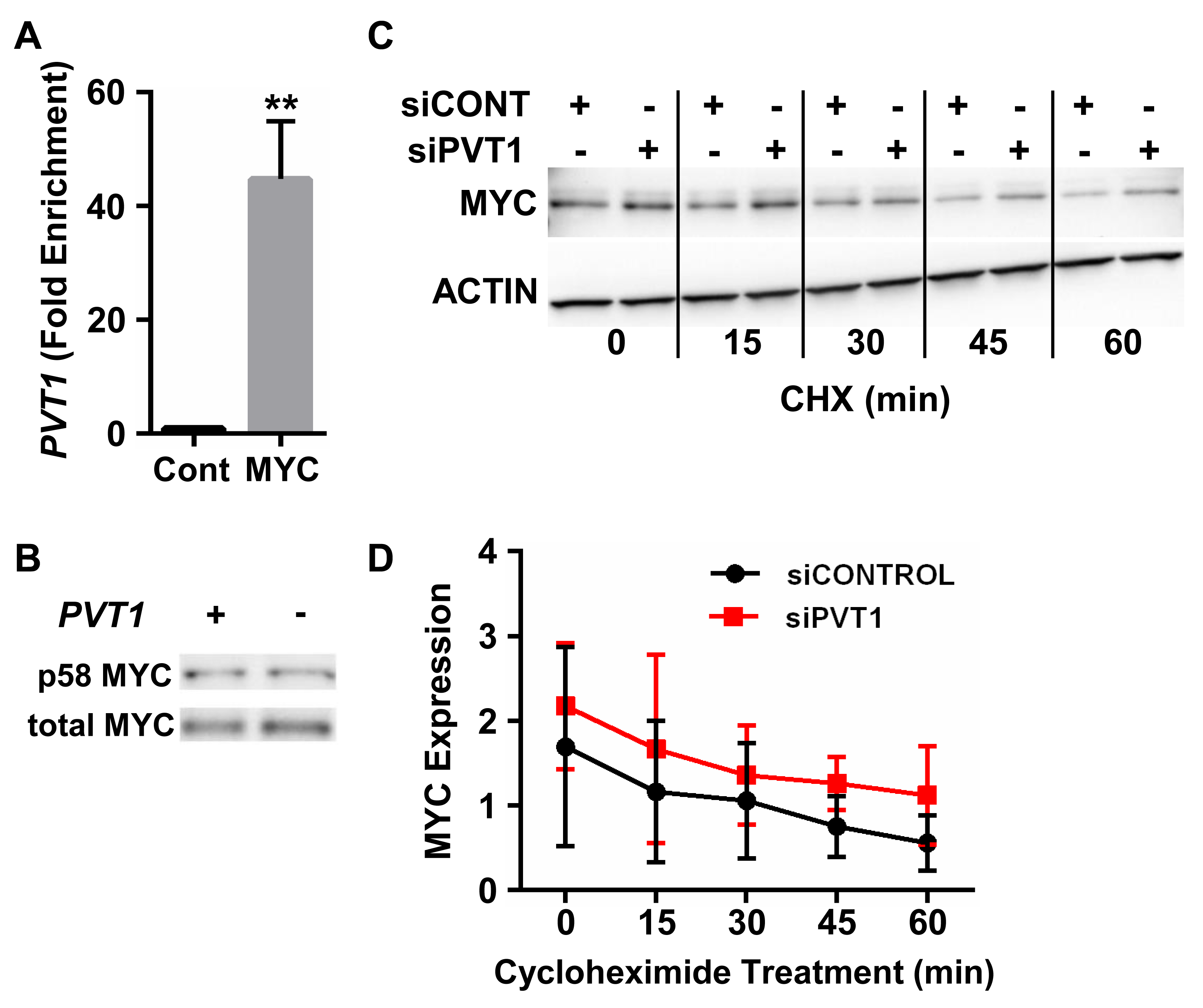

Supplement: S4 Fig — (A) PVT1 from SiHa total cell lysate immunoprecipitated with a MYC antibody, but not the negative control rabbit IgG (Cont). (B) PVT1 knockdown in SiHa cells did not significantly affect p58 phospho-MYC or total MYC protein. (C,D) Degradation of MYC protein was also not significantly affected by PVT1 knockdown. **p<0.01 (TIF) [file pone.0156274.s004.tif]

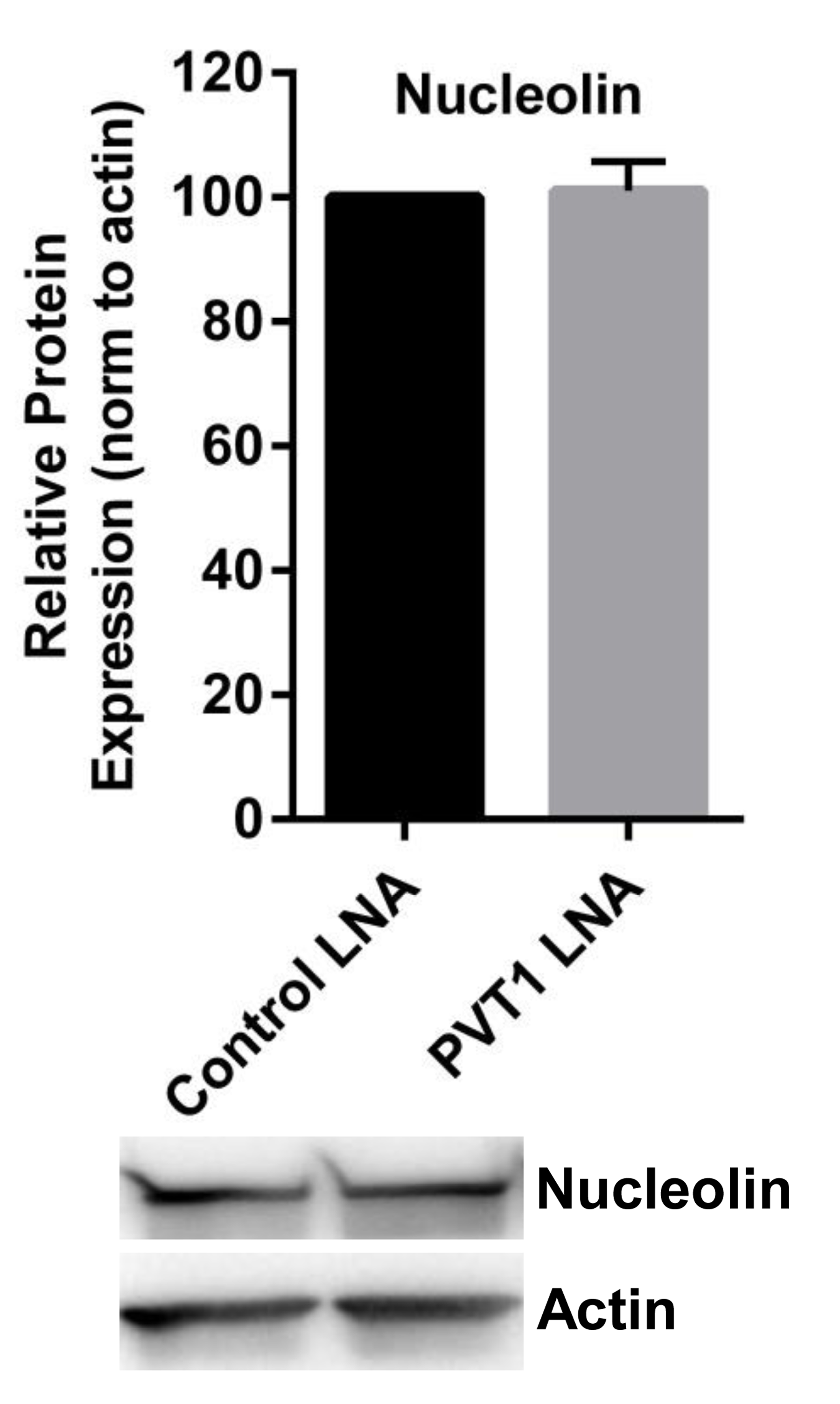

Supplement: S5 Fig — (TIF) [file pone.0156274.s005.tif]
